# A Case of Gonorrhea in the Female*Read before meeting of Medical Association of Georgia, April, 1894.

**Published:** 1894-06

**Authors:** R. J. Nunn

**Affiliations:** Savannah, Ga.


### A CASE OF GONORRHEA IN THE FEMALE.*

BY R. J. NUNN, M.D., SAVANNAH, GA.

The subject of this history was referred to me by a general practitioner for an obscure and troublesome condition of the rectum. She was a nullipara, unmarried, aged about eighteen years, and complained of a continual burning pain in the rectum, all the discharges from which were more or less purulent, and if solid were coated with pus. There was also an annoying tenesmus.

These symptoms had existed for several days before her visit to me, three days before which she began to complain of ardor urinæ, followed in two days by a vaginal discharge, which the girl called whites. There was no constitutional disturbance, but the facial expression of the patient showed marked evidence of suffering.

Here was a most unusual train of symptoms to deal with, but after revolving the matter in my mind, and recalling some former experiences in the same line, the conclusion forced itself upon me that the case was one of gonorrhea, although the social position of the patient was such that her regular medical attendant had not conceived it possible.

A careful physical examination of the patient and the secretions confirmed the opinion already formed, and the patient acknowledged

*Read before meeting of Medical Association of Georgia, April, 1894.

the probable accuracy of the diagnosis, giving a date three weeks anterior to her visit to me as the only possible time for inoculation.

The physical examination of the patient revealed a high state of inflammation of the mucous membrane of the rectum, vagina and urethra. There was, moreover, considerable endocervitis, with erosions of the os and ectropium of the endocervium. Certainly the disease had invaded the cervical endometrium; probably soon would, if it had not already extended into the body of the uterus, and might be expected to show evidences of its presence in the fallopian tubes.

The exact state of affairs at the time and the prognosis were given to the patient, and treatment begun with the result that the condition of all accessible parts was ameliorated, and the more painful and annoying symptoms disappeared; but in a month the case developed an intense peritonitis and salpingitis, accompanied with a copious purulent discharge from the os, from which she made a good recovery in ten days.

The treatment followed was copious aseptic injections and douches, followed by injections of petrolatum liquidum into the rectum and urethra. The vagina was washed with petrolatum through a speculum, and tampons saturated with it were inserted and changed daily. The endometrium was treated with tents of cocoa butter. The progress of the case was satisfactory as far as all accessible parts were concerned, but the condition of the adnexa must remain in doubt.

It was especially interesting to observe the rapid relief of the rectal symptoms which followed the injection of a couple of ounces of petrolatum, and continued from twelve to eighteen hours.

The point of interest in this history is the mode of accession, the disease first manifesting itself in the rectum, and its subsequent extension to the urethra, and lastly to the vagina.

I can recall no similar case in the literature of the disease. Indeed, the implication of the rectum is exceedingly rare, except in cases of direct inoculation, which I have been assured, and have reason to believe, was not the case in this instance.

Therapeutically, the interesting feature of the case is the treatment of it by oleaginous applications.

